# Congenital Absence of Posterior Interosseous Artery: A New Anatomic Variant of the Posterior Interosseous Artery Flap

**DOI:** 10.7759/cureus.9771

**Published:** 2020-08-15

**Authors:** Terrence Jose Jerome, Bhuvaneswari Shanmugasundaram, Thirumagal Kuppusamy Terrence

**Affiliations:** 1 Orthopaedics, Hand and Reconstructive Microsurgery, Olympia Hospital and Research Centre, Trichy, IND; 2 Pharmacology, Dhanalakshmi Medical College, Perambalur, IND; 3 Reproductive Medicine/Obstetrics and Gynecology, Olympia Hospital and Research Centre, Trichy, IND

**Keywords:** congenital absence, posterior interosseous artery

## Abstract

Soft tissue cover to the hand can be as simple as a skin graft, local, distant flaps to a complex microvascular free flap. Posterior interosseous artery (PIA) flap is a technically demanding robust flap which can be used to cover a wide range of hand and wrist defects. We report a 25-year-old lady who had severe crush injury where the posterior interosseous flap was planned to cover the dorsum of hand defects. On exploration carefully, the PIA was found to be congenitally absent. An alternative groin flap salvaged the procedure and the patient had good aesthetic and functional outcomes at the five years of follow-up.

## Introduction

The posterior interosseous artery (PIA) flap is one of the robust flaps in the reconstructive ladder for defects in the hand and wrist. Since 1986, the clinical cases and large reviews proved this flap as relatively easy to harvest and avoid sacrifices of either radial or ulnar artery [1]. Furthermore, the donor site can be primarily closed or covered with a split-thickness skin graft [2]. Several anatomical variants have been described for the PIA flap which includes the PIA found in the different fascial septum, the complete absence of anastomosis between anterior and posterior interosseous arteries, and narrowing and bilateral absence at the mid-third level [1-5]. We discuss an interesting case with a complete absence of PIA in a female patient who was planned for PIA flap and the alternate treatment offered when identified the absence intraoperatively.

## Case presentation

**Figure 1:**
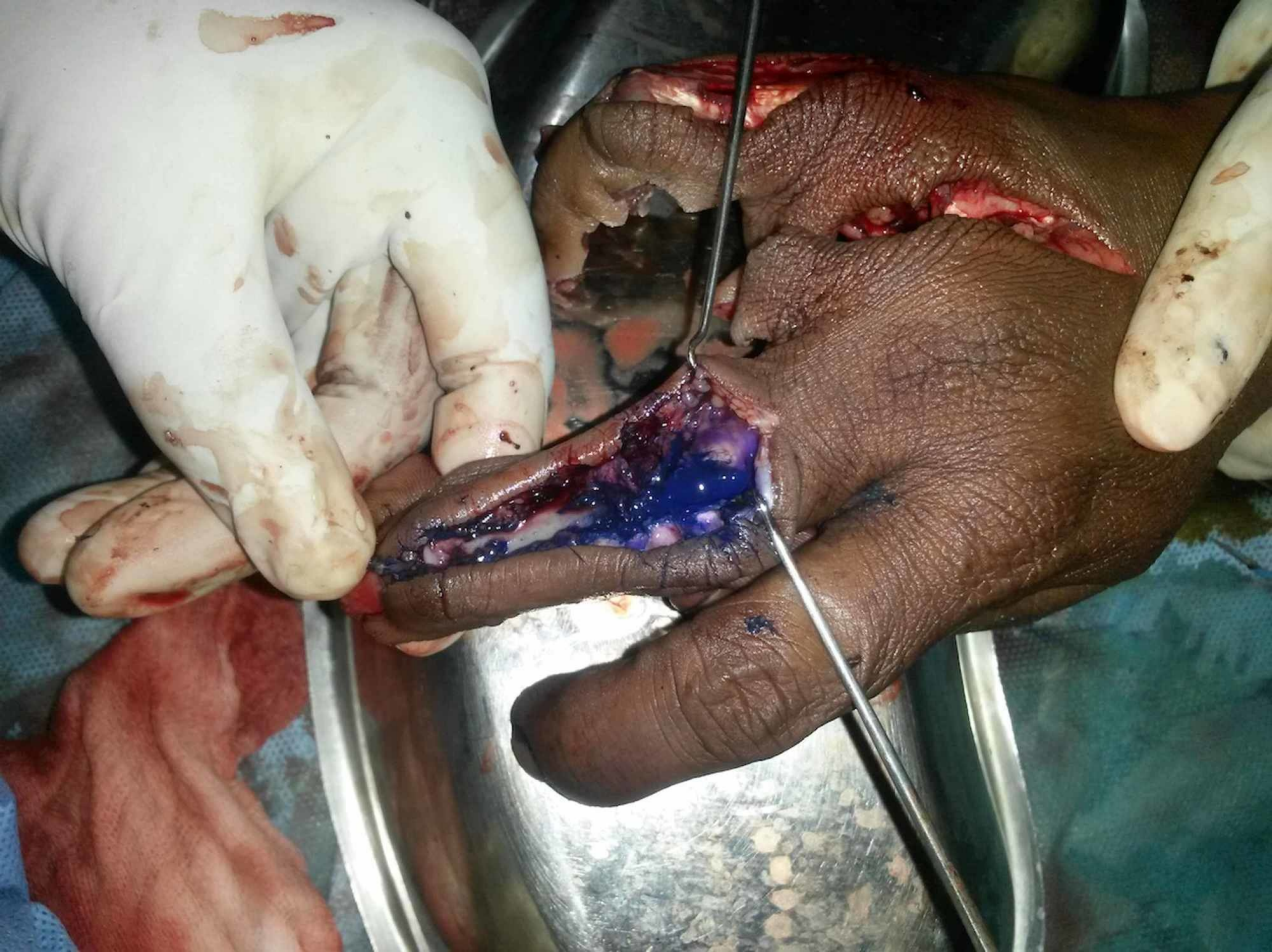
Clinical picture Severe crush injury of the right-hand involving index, middle, ring, and little finger with heavy pain contamination, flexor, extensor tendon injury, and composite soft tissue loss.

A 25-year-old mill worker who had sustained a severe crush injury to her right-hand involving index, middle, ring, and little finger both dorsal and volar sides with heavy paint contamination, extensor and flexor tendon injuries, and composite soft tissue loss (Figure 1).

**Figure 2:**
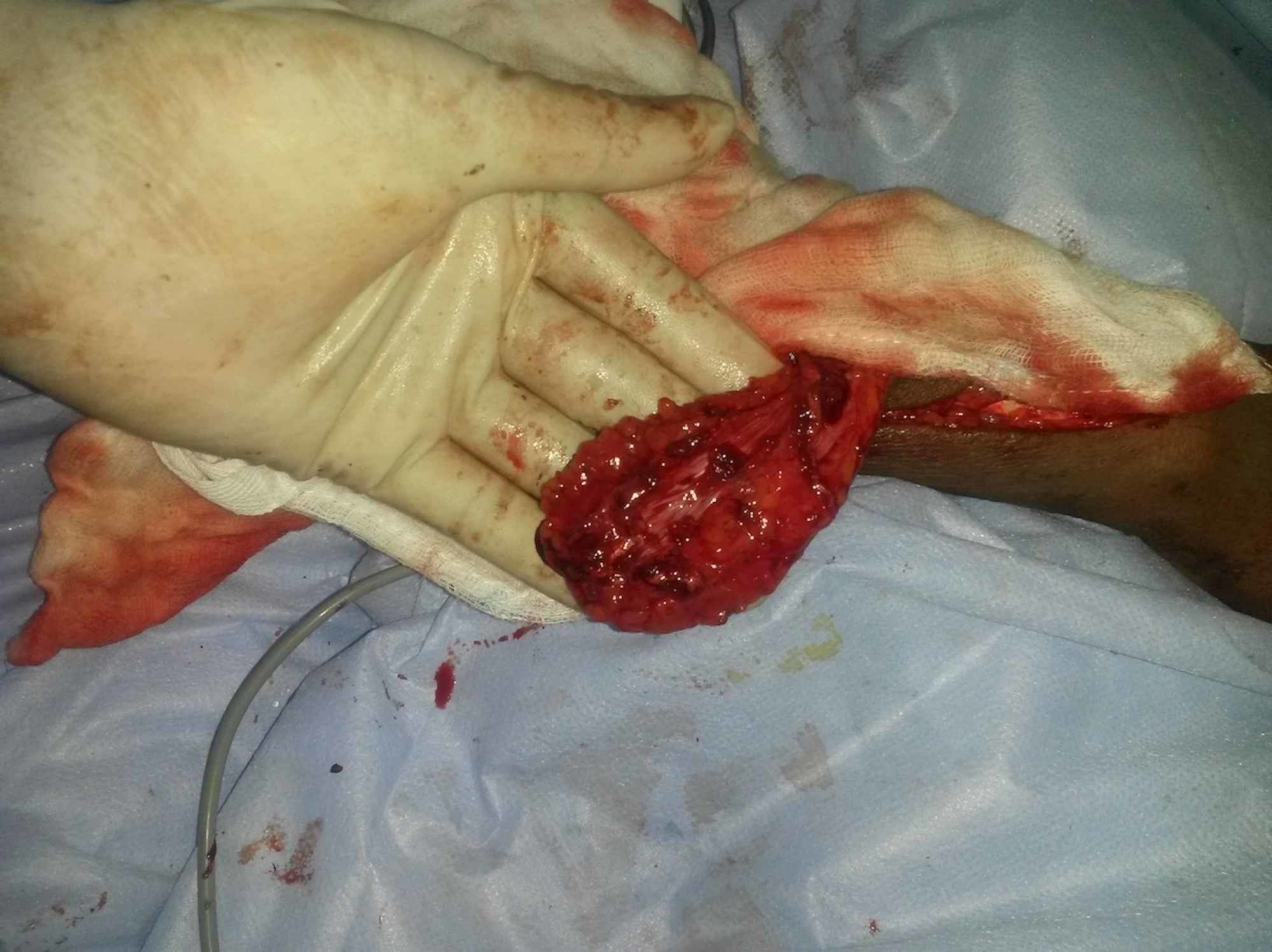
Intraoperative picture The harvest of PIA flap with the congenital absence of the PIA during the dissection. PIA: posterior interosseous artery.

Extensive wound wash and multiple wound debridement were done in the first week. Once the wound was free of paint and other contaminations, flexor, extensor tendon repair was done. At the same time, a PIA flap cover was planned for the dorsum hand defects (4 x 9 cm^2^). The preoperative Doppler signal was weak and could not find the course of the PIA, its anastomosis, which we attributed to the forearm swelling. Under the brachial block in the patient, the PIA flap dissection was performed. The PIA is relatively superficial in the distal half and proximal half is deeply situated in the septum which lies underneath the extensor digit minimi. Interestingly, the posterior interosseous vessels could not be identified anywhere in our plane of dissection (Figure 2).

We have cross-checked our surgical plane again and came to halt understanding the congenital absence of vessels in our patient. So, a split-thickness skin graft was done in the proximal flap elevated donor site, and suturing for the distal wounds was done. An alternative groin flap was raised to cover the dorsal hand defect under spinal anesthesia. The flap was divided at three weeks, and the patient made an uneventful and good esthetic and functional recovery.

## Discussion

The reconstruction of large soft tissue defects in the hand should consider a possible compromise of the remaining hand vascularization and future reconstructive options. The posterior interosseous flap offers some advantages over other available reconstructive methods since it sacrifices vessels that are not essential in maintaining hand viability [6]. Doppler testing during preoperative planning identifies the anastomosis between the posterior and anterior interosseous artery. There are three auxiliary procedures which can be designed to overcome these anatomical variations around its distal pedicle, enhancing flap reliability [7]. The PIA flap has become the first choice for soft tissue reconstruction of the first webspace, dorsal and palmar aspects of the hand, including the metacarpal-phalangeal joints and the dorsum of the thumb as well as for metacarpal reconstruction. Also, the fascio-cutaneous flaps are considered as an ideal alternative for PIA flaps to reconstruct soft-tissue defects or contractures of the thumb web and the dorsal hand because of good tissue matching [7].

The frequency of anatomic variations and the vascular complications per se is higher compared to other reverse pedicled flaps [8,9].

Table 1 has an extensive literature search and found interesting and at the same time very cautious for the operating surgeons to be aware of anatomical variability. One such rare variation is the congenital absence of the PIA which all surgeons should be enlightened. We also felt that it would have been better to carry out a complete Doppler sonographic study using a skilled investigator who ideally would have detected, and if not an arteriogram would have confirmed the absence. This is a limiting factor in our case. 

**Table 1:** Showing the literature search about the anatomic variants of PIA AIA: anterior interosseous artery, PIA: posterior interosseous artery.

Authors	Year	Cases	Anatomic variants
Masquelet and Penteado [1]	1986	4(70)	The anastomotic branch was absent in one specimen and there was the disappearance of PIA in the middle third in four of their specimens.
Costa and Soutar [2]	1988	3(22)	Termination of the PIA in the middle third of the forearm. PIA is seen in the fascial septum between the ECU and EDM until it reached the level of the ulnar head and its anastomosis to the anterior interosseous artery.
Masquelet and Penteado [1]	1986	4(70)	Absence of PIA at the mid-forearm level.
Angrigiani et al. [3]	1993	2(80)	Absence of choke anastomosis between the recurrent dorsal branch of the AIA and PIA at the level of middle third of the posterior forearm narrowing of the PIA to a very small caliber in the middle third of the forearm.
Giunta and Lukas [4]	1998	1	Hypoplastic PIA in the middle third.
Buchler and Frey [5]	1991	2(36)	The deficient anlage of the posterior interosseous vascular system distally, (1/3^rd^) 2. Anomalous origin of the relevant most proximal perforator. No PIA at the mid-forearm level.
Costa et al. [7]	2007	1(102)	Bilateral absence of PIA at the mid-forearm level.
Angrigiani et al. [3]	1993	74(80)	Narrowing of PIA in the mid-forearm.
Koch et al. [9]	2003	1(30)	PIA pierced the interosseous membrane 4 cm proximal to its usual point of entry.
Our study 2009	2020	1	Congenital absence of the entire PIA artery.

## Conclusions

Hand injuries pose a challenge in reconstruction where soft tissue loss jeopardizes the numerous underlying vital structures. The most expedient, rapid, and single-stage surgical solution to upper extremity soft-tissue coverage without hand dependency is PIA flap. Also, this flap is thin, soft pliable with better color and texture match. It can cover defects in the fingers and wrist. The preoperative handheld Doppler probe should be done to identify the distal and proximal anastomosis in all cases. In case of severe forearm swelling following injury sequelae, CT angiography may be useful. 

Congenital absence of the PIA is a rare entity where surgeons should bear this while operating. An alternate local or distant flap should be their further course of treatment if they encounter one such. 
